# The Impact of Hypertension and Metabolic Syndrome on Nitrosative Stress and Glutathione Metabolism in Patients with Morbid Obesity

**DOI:** 10.1155/2020/1057570

**Published:** 2020-09-09

**Authors:** Barbara Choromańska, Piotr Myśliwiec, Magdalena Łuba, Piotr Wojskowicz, Hanna Myśliwiec, Katarzyna Choromańska, Jacek Dadan, Anna Zalewska, Mateusz Maciejczyk

**Affiliations:** ^1^1st Department of General and Endocrine Surgery, Medical University of Bialystok, 24a M. Sklodowskiej-Curie Street, 15-276 Bialystok, Poland; ^2^Department of Dermatology and Venereology, Medical University of Bialystok, 14 Żurawia Street, 15-540 Bialystok, Poland; ^3^Department of Oral Surgery, Medical University of Gdansk, 7 Dębinki Street, 80-211 Gdansk, Poland; ^4^Experimental Dentistry Laboratory, Medical University of Bialystok, 24a M. Sklodowskiej-Curie Street, 15-274 Bialystok, Poland; ^5^Department of Hygiene, Epidemiology and Ergonomics, Medical University of Bialystok, 2c Mickiewicza Street, 15-233 Bialystok, Poland

## Abstract

In this pathbreaking study, we evaluated nitrosative stress in morbidly obese patients with and without metabolic syndrome. 62 women with class 3 obesity (BMI > 40 kg/m^2^) were divided into three subgroups: obese patients (OB), obese patients with hypertension (OB+HYP), and obese patients with metabolic syndrome (OB+MS). In comparison to the lean patients, OB had increased levels of serum myeloperoxidase (MPO), plasma nitric oxide (NO), S-nitrosothiols, and peroxynitrite (ONOO^−^), as well as nitrotyrosine, while oxidized glutathione (GSSG) rose only in OB+HYP group. Interestingly, ONOO^−^ was significantly higher in OB+HYP and OB+MS as compared to OB group, while MPO only in OB+MS group. OB+MS had greater nitrotyrosine and S-nitrosothiol values than OB+HYP. Moreover, peroxynitrite could differentiate OB from OB+HYP and OB+MS (AUC 0.9292; *p* < 0.0001; 87.5% sensitivity, 90% specificity) as well as between OB and OB+MS group (AUC 0.9125; *p* < 0.0001; 81.25% sensitivity, 83.33%). In conclusion, we showed that MPO activity, NO formation, and nitrosative damage to proteins parallel the progression of metabolic disturbances of obesity. Evaluation of ONOO^−^ concentrations may help predict the development of hypertension and metabolic syndrome in patients with morbid obesity; however, longer-term studies are required for larger numbers of patients.

## 1. Introduction

The formation of reactive oxygen (ROS) and nitrogen (RNS) species is inextricably linked to several metabolic processes in the human body [1]. Physiologically, endothelial (eNOS), inducible (iNOS), and neuronal (nNOS) nitric oxide (NO) synthases are responsible for the production of NO, which is a paracrine factor relaxing the vascular wall as well as regulating the long-term synaptic transmission, platelet aggregation, angiogenesis, arteriogenesis, and immune function [2]. Nevertheless, overproduction of RNS results in nitrosative damage to proteins and lipids [3]. Indeed, under inflammatory conditions, there is a stimulation of the inducible NOS isoform (iNOS) in response to various endogenous factors such as interferon-*γ*, interleukin 1 (IL-1), and tumor necrosis factor *α* (TNF-*α*). Interestingly, increase in iNOS activity promotes NO reaction with O_2_^−^, leading to the formation of highly reactive peroxynitrite (ONOO^−^) [4]. Additionally, myeloperoxidase (MPO) released from neutrophils binds to endothelial glycosaminoglycans and then catalyzes the production of hypochlorous acid (HOCl). HOCl in reaction with nitrogen dioxide (NO_2_) forms nitryl chloride (NO_2_Cl) which is an oxidizing compound and an apoptosis inducer [5]. Under oxidative stress conditions, there is also a decrease in the synthesis of prostacyclin (PGI_2_), a factor that relaxes blood vessels, reduces blood pressure, and inhibits platelet aggregation. Indeed, ONOO^−^ is responsible for the inhibition of prostacyclin productions, which (by nitrating tyrosine in the active center of the enzyme) reduces activity of PGI_2_ synthase [6]. ONOO^−^ also disturbs tissue remodeling by activating matrix metalloproteinases and promoting proinflammatory response. It was shown that ONOO^−^ induces expression of intercellular adhesion molecule (ICAM), macrophage-1 antigen (Mac-1) (CD11b/CD18) adhesion proteins, and P selectin. It also activates nuclear factor kappa B (NF-*κ*B) and affects expression of interleukin 8 (IL-8) in leukocytes [7].

It is well known that obesity is a major risk factor of hypertension, insulin resistance, type 2 diabetes (T2DM), and metabolic syndrome (MS). However, the pathogenesis of metabolic disturbances in obesity is very complex and still not clear. Recent studies indicate a critical role of oxidative stress in the development of obesity and its metabolic complication. It was shown that in endothelial cells highly upregulated NADPH (NOX) and xanthine (XO) oxidases result in overproduction of ONOO^−^ and decreased bioavailability of NO [8]. RNS stimulates mineralocorticoid receptor activation contributing to electrolyte imbalance and hypertension. Therefore, disturbances in prooxidant enzymes/NOS activity increase peripheral resistance and blood pressure [9, 10]. It has been suggested that hypertension contributes to metabolic syndrome by diminishing NO bioavailability [11]. A link between nitrosative stress and type 2 diabetes mellitus (T2DM) has also been described [12, 13]. Indeed, in T2DM patients, eNOS activity is overwhelming in blood vascular wall. Probably, it is caused by decreased protein kinase (Akt) activity as well as reduced phosphorylation/oxidation of cofactor tertrahydrobiopterin [14]. It has been suggested that nitrosative stress disturbs vascular reactivity of epithelial arterioles in T2DM patients [15].

In our previous studies, we have shown that disturbances in prooxidant/antioxidant imbalance generally normalize after surgical treatment of obesity [16, 17]. Nevertheless, it is still unclear whether metabolic changes accompanying obesity depend on nitrosative stress. Moreover, there are no data on the diagnostic usefulness of nitrosative stress biomarkers in patients with obesity and metabolic disturbances. Given the key role of RNS in the pathogenesis of hypertension and diabetes, we report this first study evaluating nitrosative stress in morbidly obese patients with hypertension and morbidly obese cases with metabolic syndrome, as well as morbidly obese patients without metabolic disorders.

## 2. Materials and Methods

The study was implemented in accordance with the Declaration of Helsinki and the Guidelines for Good Clinical Practice, as well as was approved by the Ethics Committee of the Medical University of Bialystok, Poland (permission: R-I-002/69/2012 and R-I-002/475/2019). All participants of the study gave their informed consent.

The study included 62 morbidly obese women with class 3 obesity (BMI > 40 kg/m^2^), aged from 28 to 56. The patients were divided into three subgroups: morbidly obese patients (OB) (*n* = 30), morbidly obese patients with hypertension (OB+HYP) (*n* = 16), and morbidly obese patients with metabolic syndrome (OB+MS) (*n* = 16). Metabolic syndrome (MS) was diagnosed according to the International Diabetes Federation guidelines [18]. In the OB+MS group, all patients had hypertension and type 2 diabetes mellitus (T2DM). According to World Health Organizations, hypertension was diagnosed if the value of systolic (SBP) and/or diastolic (DBP) blood pressure was 140/90 (mmHg) or above on two different days. Blood pressure was measured using Diagnosis UA-651 A&D Medical apparatus. Blood pressure measurements were taken on the nondominant upper arm. The cuff was put on the exposed arm, about 2-3 cm above the elbow flexion, so that it clings tightly to the shoulder. Two measurements were taken at intervals of several minutes. Morbidly obese patients with hypertension were pharmacologically treated: ACE inhibitors: lisinopril, perindopril; calcium channel blockers: amlodipine; *β* blockers: metoprolol, bispoprolol; and diuretics: indapamind, whereas patients with T2DM took metformin, insulin, and gliclazide. None of the patients was treated with captopril, *β* blockers with a nitric oxide donative action (e.g., nebivolol) nor nitrates. Morbidly obese women underwent elective bariatric surgery at the 1st Department of General and Endocrine Surgery at the University Hospital in Bialystok, Poland.

Height and body mass, as well as waist and hip circumferences were measured using standard methods. Waist circumference was measured halfway between the upper edge of the iliac crest and lower arch of the ribs and the circumference of the hips below the iliac plates through the largest protrusion of the gluteal muscles.

The lean group consisted of 30 lean healthy women (BMI < 25 kg/m^2^, aged 28 to 56) treated dentistically in Specialist Dental Clinic at the Medical University of Bialystok, Poland. Patients with reference range of blood count and biochemical blood tests (INR, creatinine, ALT, AST, Na^+^, and K^+^) were qualified to the control group.

The lean and morbidly obese women were included in the study based on the negative medical history of acute inflammatory diseases and malignancy. Patients with autoimmune diseases (ulcerative colitis, Crohn's and Hashimoto's disease); infectious diseases (HIV/AIDS, hepatitis A, B, or C); metabolic diseases, such as type 1 diabetes, mucopolysaccharidosis, gout, or osteoporosis; as well as cardiovascular (with exception of arterial hypertension in obese group), respiratory, digestive, and genitourinary system diseases were excluded from the study. Within the three months before the study, the participants declared not taking any vitamins, antioxidant supplements (including iron preparations), antibiotics, glucocorticosteroids, or nonsteroidal anti-inflammatory drugs. They also had not chronically smoked nor drunk alcohol.

### 2.1. Blood Collection

Blood samples were taken from obese and lean patients in the overnight fasting state to serum and EDTA tubes (S-Monovette SARSTEDT). In twenty-four hours prior to blood sampling, patients not had an intense physical activity. Blood samples from obese patients were collected before the bariatric surgery. The samples were centrifuged in 4°C for 10 minutes at 4,000 rpm. The serum and plasma were retained for further testing. Butylated hydroxytoluene (10 *μ*L, 0.5 M, and BHT/1 mL serum/plasma) was added to all samples to protect them against oxidation [19]. The samples were stored at -80°C until final research.

### 2.2. Laboratory Measurements

The blood counts and biochemical laboratory parameters were analyzed by using an Abbott analyzer (Abbott Diagnostics, Wiesbaden, Germany). Homeostatic model assessment index (HOMA-IR) was counted accordance with the formula [20].

### 2.3. Nitrosative Stress and Glutathione Metabolism

All reagents were purchased from Sigma-Aldrich (Nümbrecht, Germany, and/or Saint Louis, MO, USA). The absorbance was analyzed using Infinite M200 PRO Multimode Microplate Reader Tecan. All determinations were performed in duplicate samples, and results were standardized to 1 mg of total protein. The total protein content was analyzed spectrophotometrically using the bicinchoninic acid (BCA) method. Commercial diagnostic kit (Thermo Scientific PIERCE BCA Protein Assay; Rockford, IL, USA) was used according to manufacturer's instructions.

The concentration of total glutathione was determined colorimetrically at 412 nm using enzymatic reaction with 5,5′-dithiobis-(2-nitrobenzoic acid) (DTNB), NADPH, and glutathione reductase [21]. The level of total glutathione was calculated from the calibration curve for reduced glutathione (GSH). The concentration of oxidized glutathione (GSSG) was determined similarly to total glutathione assay. The difference is that before determination the samples were neutralized to pH 6-7 with 1 M chlorhydroltriethanolamine (TEA) and then incubated with 2-vinylpyridine (to inhibit glutathione oxidation). GSH level was calculated from the difference between the concentrations of total glutathione and GSSG. The redox potential was calculated based on the ratio of reduced glutathione to oxidized glutathione ([GSH]^2^/[GSSG]) [22].

The activity of serum myeloperoxidase (MPO) was analyzed spectrophotometrically using sulfanilamide hexadecyl trimethylammonium, ortho-dianisidinedihydrochloride, and hydrogen peroxide [23]. The absorbance was measured at 450 nm.

The level of plasma total nitric oxide (NO) was analyzed spectrophotometrically using sulfanilamide and NEDA·2 HCl (N-(1-naphthyl)-ethylenediamine dihydrochloride) [22, 24]. The absorbance was measured at 490 nm.

The level of plasma S-nitrosothiols was analyzed spectrophotometrically based on the reaction of the Griess reagent with Cu^2+^ ions [22, 25]. The absorbance was measured at 490 nm.

The level of plasma peroxynitrite was analyzed spectrophotometrically based on peroxynitrite-mediated nitration resulting in the formation of nitrophenol [26]. The absorbance was measured at 320 nm.

The level of plasma nitrotyrosine was analyzed spectrophotometrically by ELISA method. Commercial diagnostic kit (Immundiagnostik AG; Bensheim, Germany) was used according to manufacturer's instructions.

The precisions of these measurements, expressed as coefficients of variation (CV), were <4% (total glutathione), <4% (GSH), <5% (GSSG), <4.5% (MPO), <7% (NO), <4% (S-nitrosothiols), <3% (peroxynitrite), and <4% (nitrotyrosine).

### 2.4. Statistical Analysis

GraphPad Prism 8.3.0 for MacOS (GraphPad Software, Inc. La Jolla, USA) was used to statistical data processing. The normality of the distribution was assessed using the Shapiro–Wilk test. Characteristics of data sets were presented by the method of descriptive analysis. For comparison of quantitative variables, ANOVA Kruskal–Wallis and Dunn's test were used. The statistical significance was set at *p* < 0.05. Multiplicity adjusted *p* value was also calculated. The association between nitrosative stress biomarkers was evaluated using the Spearman rank correlation. The diagnostic usefulness of assessed biomarkers was determined by receiver operating characteristic (ROC) analysis. The number of patients was calculated *a priori* based on our previous experiment. The power of the test was 0.9 (ClinCalc online calculator).

## 3. Results

Not surprisingly, we observed higher values of BMI and WHR in each group of patients with obesity, compared with lean patients (Table 1). Systolic (SBP) and diastolic (DBP) blood pressures were greater in OB+HYP and OB+MS than in the control group. Additionally, SBP was higher in OB+MS patients than OB ones. Glucose, insulin, HOMA-IR, total cholesterol, uric acid, and fibrinogen had greater values, whereas HDL was diminished in each obese study group in comparison to the lean patients. Moreover, we noticed greater LDL, CRP, and WBC in OB+HYP and OB+MS patients than in the controls. LDL concentrations were higher in OB+HYP and OB+MS than OB group (Table 1).

### 3.1. Plasma Total Glutathione

**Figure 1:**
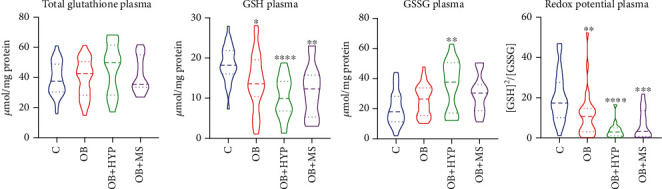
Violin plots of plasma total glutathione, GSH, GSSG, and redox potential of the control, morbid obesity (OB), morbid obesity with hypertension (OB+HYP), and morbid obesity with metabolic syndrome (OB+MS). ^∗^*p* < 0.05, ^∗∗^*p* < 0.01, ^∗∗∗^*p* < 0.001, and ^∗∗∗∗^*p* < 0.0001 indicate significant differences from the control; reduced glutathione (GSH), oxidized glutathione (GSSG).

There were no statistically significant differences in the plasma total glutathione between morbidly obese study groups and the lean controls (Figure 1).

### 3.2. Plasma Reduced Glutathione (GSH)

The plasma concentration of GSH was lower in each morbidly obese study group: OB (-22%, *p* = 0.0137), OB+HYP (-45%, *p* < 0.0001), and OB+MS (-33%, *p* = 0.0019) as compared to the controls (Figure 1).

### 3.3. Plasma Oxidized Glutathione (GSSG)

We found markedly higher plasma concentrations of GSSG only in OB+HYP (+50%, *p* = 0.0025) patients in comparison with lean ones (Figure 1).

### 3.4. Redox Potential

Plasma redox status of OB (-35%, *p* = 0.006), OB+HYP (-83%, *p* < 0.0001), and OB+MS (-81%, *p* = 0.0005) was significantly diminished as compared to the control group (Figure 1).

### 3.5. Serum Myeloperoxidase (MPO)

**Figure 2:**
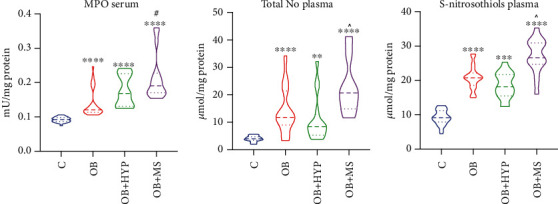
Violin plots of serum MPO, plasma total NO and plasma S-nitrosothiols of the control, morbid obesity (OB), morbid obesity with hypertension (OB+HYP), and morbid obesity with metabolic syndrome (OB+MS). ^∗∗^*p* < 0.01, ^∗∗∗^*p* < 0.001, and ^∗∗∗∗^*p* < 0.0001 indicate significant differences from the control; ^#^*p* < 0.05 indicates significant differences from the morbid obesity (OB); ^*p* < .0.05 indicates significant differences from the morbid obesity with hypertension (OB+HYP); myeloperoxidase (MPO), total nitric oxide (NO).

We observed significantly increased activities of serum MPO in each obese study group: OB (+30%, *p* < 0.0001), OB+HYP (+85%, *p* < 0.0001), and OB+MS (+107%, *p* < 0.0001) in comparison with the lean controls. Additionally, activity of serum MPO was higher in OB+MS (+58%, *p* = 0.0253) subgroup than OB ones (Figure 2).

### 3.6. Total Plasma Nitric Oxide (NO)

NO total plasma concentration of OB (+200%, *p* < 0.0001), OB+HYP (+110%, *p* = 0.0031), and OB+MS (+425%, *p* < 0.0001) was statistically higher as compared to the controls. Interestingly, OB+MS patients had significantly greater total plasma concentration of NO (+150%, *p* = 0.0215) compared to OB+HYP (Figure 2).

### 3.7. Plasma S-Nitrosothiols

We noticed markedly higher plasma concentration of S-nitrosothiolos in OB (+128%, *p* < 0.0001), OB+HYP (+96%, *p* = 0.0002), and OB+MS (+193%, *p* < 0.0001) groups than the lean patients. Moreover, plasma concentration of S-nitrosothiolos was greater in OB+MS (+50%, *p* = 0.0177) in comparison with OB+HYP patients (Figure 2).

### 3.8. Plasma Peroxynitrite

**Figure 3:**
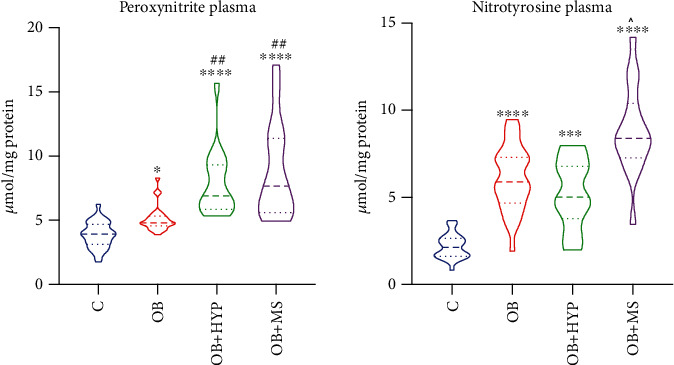
Violin plots of plasma peroxynitrite and nitrotyrosine of the control, morbid obesity (OB), morbid obesity with hypertension (OB+HYP), and morbid obesity with metabolic syndrome (OB+MS). ^∗^*p* < 0.05, ^∗∗∗^*p* < 0.001, and ^∗∗∗∗^*p* < 0.0001 indicate significant differences from the control; ^##^*p* < 0.01 indicates significant differences from the morbid obesity (OB); ^*p* < .0.05 indicates significant differences from the morbid obesity with hypertension (OB+HYP).

We found significantly greater plasma concentration of ONOO^−^ in each group of obese patients: OB (+23%, *p* = 0.0148), OB+HYP (+77%, *p* < 0.0001), and OB+MS (+97%, *p* < 0.0001) as compared to the lean controls. Further on, we observed elevated peroxynitrite concentrations in OB+HYP (+44%, *p* = 0.0011) and OB+MS (+60%, *p* = 0.0009) patients compared to OB ones (Figure 3).

### 3.9. Plasma Nitrotyrosine

Plasma nitrotyrosine concentration was increased in OB (+180%, *p* < 0.0001), OB+HYP (+138%, *p* = 0.0007), and OB+MS (+300%, *p* < 0.0001) patients in comparison with the controls. Interestingly, we noticed that OB+MS (+68%, *p* = 0.0206) patients had higher concentration of nitrotyrosine than OB+HYP patients (Figure 3).

### 3.10. Correlations

Correlations between the analyzed nitrosative stress parameters and glutathione and clinical parameters are presented in the supplementary material (Table 1).

In morbidly obese patients, serum MPO correlated positively with ONOO^−^ (*R* = 0.515; *p* < 0.0001), nitrotyrosine (*R* = 0.303; *p* = 0.017), glucose (*R* = 0.334; *p* = 0.008), cholesterol (*R* = 0.351; *p* = 0.005), and SBP (*R* = 0.366; *p* = 0.003). Plasma NO was associated with S-nitrosothiols (*R* = 0.305; *p* = 0.016), nitrotyrosine (*R* = 0.338; *p* = 0.007), and TG (*R* = 0.366; *p* = 0.004). A positive correlation was also observed between plasma S-nitrosothiols and glucose (*R* = 0.398; *p* = 0.001). Further on, plasma ONOO^−^ positively correlated with glucose (*R* = 0.433; *p* = 0.00044), HOMA-IR (*R* = 0.307; *p* = 0.01513), UA (*R* = 0.358; *p* = 0.00423), cholesterol (*R* = 0.453; *p* = 0.00021), LDL (*R* = 0.47; *p* = 0.00013), TG (*R* = 0.335; *p* = 0.00896), CRP (*R* = 0.321; *p* = 0.01095), and SBP (*R* = 0.359; *p* = 0.00413). The negative correlations were observed between GSH and BMI (*R* = −0.4; *p* = 0.001), GSH and LDL (*R* = −0.326; *p* = 0.01), and redox potential and BMI (*R* = −0.312; *p* = 0.015), as well as redox potential and LDL (*R* = −0.417; *p* = 0.001).

### 3.11. ROC Analysis

An important part of the study was also the analysis of diagnostic usefulness of the assessed nitrosative stress biomarkers. The results of ROC analysis are presented in Table 3. Interestingly, plasma peroxynitrite with high sensitivity and specificity differentiates obese from obese and hypertensive patients as well as OB group from OB+MS. Moreover, plasma NO and nitrotyrosine have a high diagnostic value in differentiating patients with obesity and hypertension from those with obesity and metabolic syndrome (Table 2).

## 4. Discussion

The adipose tissue is an important organ with multiple regulatory functions [27]. Metabolic abnormalities within adipose tissue can contribute to obesity complications including excess adipokine secretion: visfatin, leptin, and resistin, but also TNF-*α* as and plasminogen activator inhibitor-1 (PAI-1) [28–31]. Recent studies indicate that oxidative and nitrosative stress also play a critical role in the development of metabolic diseases [9, 14, 16]. Nevertheless, the knowledge about nitrosative stress in obese patients is still limited. This is the first study evaluating nitrosative stress in morbidly obese patients with hypertension and metabolic syndrome in comparison with obese patients without those complications.

Interestingly, we observed the highest activity of serum MPO in OB+MS subgroup. MPO is a marker of inflammation released by neutrophils and monocytes during activation of inflammatory cells. It not only initiates and maintains acute and chronic inflammation, but also intensifies oxidative and nitrosative stress leading to tissue injury [32–34]. Enhanced expression of MPO in neutrophils has been demonstrated to be associated with inflammation in the adipose tissue. In our study, MPO activity was higher in OB+MS patients than the OB ones. This indicates intensification of the inflammation in obese patients with metabolic syndrome. The positive correlation between MPO and glucose and between cholesterol and SBP is also noteworthy. Indeed, in the early stage of obesity, neutrophils infiltrating adipose tissue lead to release of different substances like free radicals, TNF-*α*, and MPO [35]. MPO initiates an acute inflammatory response and promotes chronic inflammation through the generation of prooxidants (HOCl and tyrosyl radicals) [36, 37].

NO has a crucial role in the regulation of body composition, systemic metabolism, and insulin sensitivity [38]. NOS inhibitors were observed to promote weight loss, regulate the secretion of insulin, and improve peripheral insulin sensitivity [39, 40]. In the present study, we found significantly higher concentration of plasma NO in OB, OB+HYP, and OB+MS groups. Bulotta et al. [41] showed that inflammatory cytokines (mainly TNF-*α*) increase NOS expression in HeLa Tet-off cells. NO synthase can be activated via ceramide-dependent way [41] and sphingomyelinase/sphingosine-1-phosphate pathways [42] as well as phosphatidyl inositol 3-kinase–protein kinase (PI3K–Akt) signaling [43]. In our previous study, we found a significant link between ceramide content in visceral fatty tissue and metabolic syndrome [31]. In the present study, we evaluated concentration of total plasma NO; however, we did not assess the source of NO production. Nevertheless, it had previously been shown that NO, generated by eNOS3 and nNOS, regulated blood pressure, while iNOS-derived NO played a critical role in inflammatory processes. Indeed, iNOS is expressed in various cells in response to inflammatory signals and has the highest capacity to produce NO [44]. Therefore, chronic inflammation in morbidly obese patients may be responsible for higher amount of NO associated with iNOS activity. This may also explain the increase in NO concentration in patients with metabolic syndrome compared to the OB+HYP group, wherein patients were reported to have endothelial dysfunction. However, we must admit that the impact of antihypertensive and antidiabetic drugs on concentration of plasma NO in our study cannot be excluded. In our experiment, most of the patients with OB+MS subgroup were treated with metformin, which restores NO bioavailability [45].

The basic mechanism decreasing NO bioavailability is a direct reaction of NO with O_2_^−^. The resulting ONOO^−^ is a strong oxidizing agent with a broad spectrum for damage to biomolecules [46, 47]. It reacts with both nucleic acids, amino acids (tyrosine, methionine, and tryptophan cysteine), and lipids (cell membrane phospholipids, LDL lipoproteins), as well as various antioxidants, changing their structure and functions [48]. ONOO^−^ can also oxidize thiol groups of enzymes and signaling proteins as well as proteins involved in energy cell metabolism (e.g., succinate dehydrogenase and fumarate reductase) [5, 49]. Nitrotyrosine, a marker of ONOO^−^-mediated protein damage, is responsible for the NO-related cytotoxicity [46, 47, 50, 51]. Higher level of 3-nitrotyrosine has been demonstrated in various systemic diseases including coronary artery disease, in myocytes of hypertensive patients, and blood vessels with atherosclerotic lesions [52–54]. In this study, we found higher concentration of peroxynitrite and nitrotyrosine in all obese subgroups as compared to the lean controls. Interestingly, peroxynitrite concentration in OB+HYP and OB+MS patients was higher than OB ones, whereas nitrotyrosine was greater in OB+MS subgroup than OB+HYP.

S-nitrosothiols, formed by the reaction of RNS with cellular thiols, are another biomarker used in the assessment of nitrosative damage [47]. We showed increased concentration of S-nitrosothiols in all obese subgroups than the controls. Moreover, S-nitrosothiols were greater in OB+MS subgroup than OB+HYP. In biological systems, S-nitrosothiols serve as reservoirs and NO transporting factors [55]. The formation of S-nitrosothiols also protects against the cellular toxicity of ONOO^−^, O_2_^−^, and NO_2_^−^ as well as NO. In addition to higher production of S-nitrosothiols, we found that plasma GSH was significantly lower in morbidly obese cases as compared to the controls. This is not surprising, because excessive NOS induction leads to endotoxic damage accompanied by a decrease in GSH level [56]. Interestingly, we also showed an increase in glutathione oxidation (↑GSSG) in patients with obesity and hypertension. It is suggested that diminishing GSH level increases the sensitivity of cells to RNS cytotoxicity. This is due to the special properties of glutathione. Not only does it sweep away oxygen free radicals, but also regenerate other oxidized antioxidants and take part in the repair of oxidative stress-damaged biomolecules as well as keep thiol groups of proteins in a reduced state. Decreased redox potential (GSH/GSSG ratio) confirms the occurrence of oxido-reductive imbalance in patients with obesity, hypertension, and metabolic syndrome.

Nowadays, the usefulness of oxidative/nitrosative stress parameters as diagnostic markers has been emphasized. Indeed, redox biomarkers have proved useful in metabolic diseases such as obesity, insulin resistance, type 2 diabetes, hypertension, and metabolic syndrome [10–12, 46, 57–59]. In this study, we investigated whether biomarkers of nitrosative stress could differentiate morbidly obese patients with hypertension and/or metabolic syndrome from those with obesity only. For this purpose, we used receiver operating characteristic (ROC) analysis. We observed that plasma ONOO^−^ can differentiate obese patients from those with obesity and hypertension (AUC 0.9292; *p* < 0.0001; 87.5% sensitivity, 90% specificity) as well as between obese subjects and OB+MS group (AUC 0.9125; *p* < 0.0001; 81.25% sensitivity, 83.33%). Thus, can plasma ONOO^−^ be a predictor of metabolic complications development in obesity? Although our study does not fully explain the underlying mechanism, we observe a clear-cut association between ONOO^−^ level and progression of metabolic complications of obesity. Plasma ONOO^−^ was also positively correlated with plasma glucose, insulin, HOMA-IR, LDL, total cholesterol, TG, UA, CRP, and fibrinogen, as well as SBP. Interestingly, S-nitrosothiols have also high diagnostic value differentiating OB+HYP patients with OB+MS ones with 81.25% sensitivity and 81.25% specificity (AUC 0.9141; *p* < 0.0001). Plasma S-nitrosothiols were also positively associated with plasma glucose in morbidly obese patients. To confirm clinical relevance, further research is needed on a larger population of patients. Long-term observation of patients is also desirable.

Our study has several limitations. We cannot eliminate the influence of hypotensive and antidiabetic drugs on the evaluated redox biomarkers. For uniformity, exclusively women were included in the study, since they constitute the majority of bariatric patients. However, as we have shown in our previous studies, the redox balance does not generally depend on gender [60]. Nitrosative stress is inextricably linked to increased production of cytokines and chemokines, which were not assessed in this study.

In conclusion, this is the first study assessing nitrosative stress in patients with obesity complicated by hypertension and metabolic syndrome, as compared with uncomplicated obesity. It has shown that the progression of metabolic disturbances, accompanying obesity, is paralleled by increased MPO activity, NO formation, and nitrosative protein damage. Evaluation of ONOO^−^ concentrations may help predict the development of hypertension and metabolic syndrome in patients with morbid obesity. In patients with morbid obesity, antioxidant supplementation should be considered.

## Figures and Tables

**Table 1:** Clinical characteristics of the control, morbid obesity (OB), morbid obesity with hypertension (OB+HYP), and morbid obesity with metabolic syndrome (OB+MS). Data given as median (lower and upper confidence limit), ^∗^*p* < 0.05, ^∗∗^*p* < 0.01, ^∗∗∗^*p* < 0.001, and ^∗∗∗∗^*p* < 0.0001 indicate significant differences from the control; ^#^*p* < 0.05, ^##^*p* < 0.01, and ^###^*p* < 0.001 indicate significant differences from the morbid obesity (OB); alanine transaminase (ALT), aspartate transaminase (AST), body mass index (BMI), C-reactive protein (CRP), creatinine (Crea), diastolic blood pressure (DBP), high-density lipoprotein (HDL), hemoglobin (HGB), homeostatic model assessment of insulin resistance (HOMA-IR), low-density lipoprotein (LDL), red blood cell count (RBC), systolic blood pressure (SBP), triacylglycerol (TG), uric acid (UA), white blood cell count (WBC), waist-hip ratio (WHR).

	C *n* = 30	OB *n* = 30	OB+HYP *n* = 16	OB+MS *n* = 16	ANOVA
Age	46 (39-49)	43 (37-49)	47 (43-49)	50 (44-52)	0.0785
BMI (kg/m^2^)	23 (23-23)	45^∗∗∗∗^ (42-46)	47^∗∗∗∗^ (43-53)	46^∗∗∗∗^ (44-50)	<0.0001
WHR	0.72 (0.71-0.72)	0.96^∗∗∗∗^ (0.94-0.99)	0.99^∗∗∗∗^ (0.96-1)	0.97^∗∗∗∗^ (0.96-0.99)	<0.0001
SBP (mmHg)	120 (120-120)	120 (120-130)	133^∗∗∗^ (120-140)	140^∗∗∗∗^^###^ (130-140)	<0.0001
DBP (mmHg)	80 (80-80)	80 (80-90)	90^∗∗^ (80-90)	90^∗∗^ (80-90)	0.0002
Glucose (mg/dL)	77 (75-80)	97^∗∗∗∗^ (92-100)	103^∗∗∗∗^ (95-110)	119^∗∗∗∗^^###^ (105-133)	<0.0001
Insulin (*μ*IU/mL)	7.6 (7.3-7.8)	18^∗∗∗∗^ (15-18)	20^∗∗∗∗^ (16-26)	22^∗∗∗∗^ (20-25)	<0.0001
HOMA-IR	1.4 (1.4-1.6)	4.1^∗∗∗∗^ (3.6-4.4)	4.8^∗∗∗∗^ (3.7-6.8)	6.2^∗∗∗∗^ (5.6-7)	<0.0001
Cholesterol (mg/dL)	175 (170-178)	195^∗^ (180-201)	215^∗∗∗∗^ (195-233)	217^∗∗∗∗^^#^ (183-231)	<0.0001
LDL (mg/dL)	118 (116-120)	124 (114-133)	145^∗∗∗∗^^###^ (139-170)	144^∗∗∗∗^^##^ (128-158)	<0.0001
TG (mg/dL)	135 (131-138)	131 (110-145)	140 (127-187)	151 (122-191)	0.1981
HDL (mg/dL)	60 (55-62)	49^∗∗∗^ (41-55)	47^∗∗^ (36-55)	44^∗∗∗^ (39-55)	<0.001
ALT (IU/L)	23 (21-27)	24 (19-30)	25 (17-34)	31^∗^ (26-44)	0.0352
UA (mg/dL)	3.9 (3.6-4.3)	6^∗∗∗∗^ (5.3-6.5)	7.2^∗∗∗∗^ (5.5-9.1)	7^∗∗∗∗^ (6-7.8)	<0.0001
Urea (mg/dL)	23 (20-28)	27 (24-32)	32^∗^ (27-37)	28 (25-34)	0.0116
Crea (mg/dL)	0.71 (0.69-0.75)	0.73 (0.71-0.75)	0.72 (0.69-0.77)	0.72 (0.64-0.77)	0.6677
CRP (mg/L)	5.5 (5.3-5.6)	8.8 (4-11)	12^∗∗∗^ (7.8-13)	12^∗∗∗^ (6.3-14)	<0.001
Fibrinogen (mg/dL)	344 (308-362)	422^∗∗∗∗^ (403-457)	432^∗∗∗∗^ (409-500)	441^∗∗∗∗^ (426-484)	<0.0001
WBC (10^3^/*μ*L)	7.5 (6.8-7.8)	8.3 (7.1-9)	9.9^∗∗∗^ (8.2-11)	8.8^∗^ (7.3-9.9)	0.0002
RBC (10^6^/*μ*L)	4.6 (4.5-4.8)	4.7 (4.4-5)	4.5 (4.2-4,8)	4.6 (4.4-4.9)	0.5193
HGB (g/dL)	14 (13-14)	13 (13-14)	13 (13-14)	14 (13-14)	0.4168
PLT (10^3^/*μ*L)	292 (278-300)	286 (247-306)	263 (220-322)	265 (191-312)	0.4496
Hypertension *n* (%)	0 (0)	0 (0)	16 (100)	16 (100)	ND
Type 2 diabetes *n* (%)	0 (0)	0 (0)	0 (0)	16 (100)	ND

**Table 2:** Area under the curve (AUC) of nitrosative stress biomarkers and glutathione between patients with morbid obesity (OB) and morbid obesity and hypertension (OB+HYP); patients with morbid obesity (OB) and morbid obesity and metabolic syndrome (OB+MS) as well as patients with morbid obesity and hypertension (OB+HYP) and morbid obesity and metabolic syndrome (OB+MS); reduced glutathione (GSH), oxidized glutathione (GSSG), myeloperoxidase (MPO) nitric oxide (NO).

	OB vs. OB+HYP							
	AUC	95% CI	*p* value	Cutoff	Sensitivity %	95% CI	Specificity %	95% CI
MPO	0.8021	0.6770 to 0.9271	0.0008	>0.1358	75	50.50% to 89.82%	76.67	59.07% to 88.21%
NO	0.6583	0.4810 to 0.8357	0.0796	< 10.31	75	50.50% to 89.82%	73.33	55.55% to 85.82%
S-nitrosothiols	0.6375	0.4573 to 0.8177	0.128	<20.24	62.5	38.64% to 81.52%	63.33	45.51% to 78.13%
Peroxynitrite	0.9292	0.8568 to 1.000	<0.0001	>5.705	87.5	63.98% to 97.78%	90	74.38% to 96.54%
Nitrotyrosine	0.6167	0.4442 to 0.7891	0.1965	<5.572	56.25	33.18% to 76.90%	60	42.32% to 75.41%
Total glutathione	0.619	0.4355 to 0.8024	0.1853	>46.46	62.5	38.64% to 81.52%	67.74	50.14% to 81.43%
GSSG	0.7036	0.5191 to 0.8882	0.0234	>31.56	68.75	44.40% to 85.84%	64.52	46.95% to 78.88%
GSH	0.6573	0.5000 to 0.8145	0.0799	<12.39	62.5	38.64% to 81.52%	64.52	46.95% to 78.88%
Redox potential	0.7333	0.5848 to 0.8819	0.011	<3.618	66.67	41.71% to 84.82%	74.19	56.75% to 86.30%
	OB vs. OB+MS							
	AUC	95% CI	*p* value	Cutoff	Sensitivity %	95% CI	Specificity %	95% CI
MPO	0.8563	0.7481 to 0.9644	<0.0001	>0.1705	75	50.50% to 89.82%	76.67	59.07% to 88.21%
NO	0.7833	0.6525 to 0.9141	0.0017	>15.72	75	50.50% to 89.82%	73.33	55.55% to 85.82%
S-nitrosothiols	0.8458	0.7109 to 0.9807	0.0001	>24.02	81.25	56.99% to 93.41%	83.33	66.44% to 92.66%
Peroxynitrite	0.9125	0.8317 to 0.9933	<0.0001	>5.481	81.25	56.99% to 93.41%	83.33	66.44% to 92.66%
Nitrotyrosine	0.8125	0.6742 to 0.9508	0.0005	>7.339	75	50.50% to 89.82%	76.67	59.07% to 88.21%
Total glutathione	0.5484	0.3708 to 0.7260	0.59	>40.60	43.75	23.10% to 66.82%	45.16	29.16% to 62.23%
GSSG	0.5948	0.4208 to 0.7687	0.2913	>30.06	62.5	38.64% to 81.52%	61.29	43.82% to 76.27%
GSH	0.5948	0.4232 to 0.7663	0.2913	<12.78	62.5	38.64% to 81.52%	61.29	43.82% to 76.27%
Redox potential	0.6215	0.4418 to 0.8012	0.1855	<6.495	60	35.75% to 80.18%	58.06	40.77% to 73.58%
	OB+HYP vs. OB+MS							
	AUC	95% CI	P value	Cutoff	Sensitivity %	95% CI	Specificity %	95% CI
MPO	0.6523	0.4540 to 0.8507	0.1416	>0.1743	62.5	38.64% to 81.52%	62.5	38.64% to 81.52%
NO	0.8594	0.7158 to 1.000	0.0005	>14.11	81.25	56.99% to 93.41%	81.25	56.99% to 93.41%
S-nitrosothiols	0.9141	0.8106 to 1.000	<0.0001	>22.41	81.25	56.99% to 93.41%	81.25	56.99% to 93.41%
Peroxynitrite	0.5391	0.3292 to 0.7489	0.7063	>7.290	56.25	33.18% to 76.90%	56.25	33.18% to 76.90%
Nitrotyrosine	0.8945	0.7781 to 1.000	0.0001	>6.945	81.25	56.99% to 93.41%	81.25	56.99% to 93.41%
Total glutathione	0.5703	0.3595 to 0.7811	0.4975	<44.41	62.5	38.64% to 81.52%	62.5	38.64% to 81.52%
GSSG	0.6367	0.4351 to 0.8384	0.1871	<32.50	62.5	38.64% to 81.52%	62.5	38.64% to 81.52%
GSH	0.5586	0.3551 to 0.7620	0.5718	>11.52	56.25	33.18% to 76.90%	56.25	33.18% to 76.90%
Redox potential	0.5778	0.3630 to 0.7926	0.4679	>3.081	53.33	30.12% to 75.19%	53.33	30.12% to 75.19%

## Data Availability

The article contains complete data used to support the findings of this study.

## Supplementary Materials

Supplementary Materials Table 1: correlations between the analyzed nitrosative stress parameters and glutathione and clinical parameters in patients with morbid obesity (OB), with morbid obesity and hypertension (OB+HYP) and patients with morbid obesity and metabolic syndrome (OB+MS).
