# Supplementary material for: The Impact of Hypertension and Metabolic Syndrome on Nitrosative Stress and Glutathione Metabolism in Patients with Morbid Obesity
Source: Oxid Med Cell Longev. 2020 Sep 9;2020:1057570. doi: 10.1155/2020/1057570 (PMC7501544; doi:10.1155/2020/1057570)
Supplement: Supplementary Materials — Table 1: correlations between the analyzed nitrosative stress parameters and glutathione and clinical parameters in patients with morbid obesity (OB), with morbid obesity and hypertension (OB+HYP) and patients with morbid obesity and metabolic syndrome (OB+MS). [file 1057570.f1.docx]

Table 1. Correlations between the analyzed nitrosative stress parameters and glutathione and clinical parameters in patients with morbid obesity (OB), with morbid obesity and hypertension (OB+HYP) and patients with morbid obesity and metabolic syndrome (OB+MS);, body mass index (BMI)C-reactive protein (CRP), creatinine (Crea), diastolic blood pressure (DBP), reduced glutathione (GSH), oxidized glutathione (GSSG), high-density lipoprotein (HDL), hemoglobin (HGB), homeostatic model assessment of insulin resistance (HOMA-IR), low-density lipoprotein (LDL), myeloperoxidase (MPO) nitric oxide (NO), red blood cell count (RBC), systolic blood pressure (SBP), triacylglycerol (TG), uric acid (UA), white blood cell count (WBC), waist-hip ratio (WHR)

|  | Total glutathione | GSH | GSSG | Redox potential | MPO | Total NO | S-nitrosothiols | Peroxynitrite | Nitrotyrosine |
| --- | --- | --- | --- | --- | --- | --- | --- | --- | --- |
| Total glutathione | 1 | **0.295^*^** | **0.888^****^** | -0.044 | 0.034 | 0.056 | 0.25 | 0.081 | -0.158 |
| GSH | **0.295^*^** | 1 | -0.135 | **0.907^****^** | -0.078 | 0.084 | 0.118 | -0.088 | -0.001 |
| GSSG | **0.888^****^** | -0.135 | 1 | **-0.448^****^** | 0.088 | -0.018 | 0.196 | 0.123 | -0.149 |
| Redox potential | -0.044 | **0.907^****^** | **-0.448^***^** | 1 | -0.149 | 0.13 | 0.008 | -0.157 | 0.004 |
| MPO | 0.034 | -0.078 | 0.088 | -0.149 | 1 | 0.14 | 0.119 | **0.515^****^** | **0.303^*^** |
| Total NO | 0.056 | 0.084 | -0.018 | 0.13 | 0.14 | 1 | **0.305^*^** | **0.292^*^** | **0.338^**^** |
| S-nitrosothiols | 0.25 | 0.118 | 0.196 | 0.008 | 0.119 | **0.305^*^** | 1 | 0.215 | 0.236 |
| Peroxynitrite | 0.081 | -0.088 | 0.123 | -0.157 | **0.515^****^** | **0.292^*^** | 0.215 | 1 | **0.256^*^** |
| Nitrotyrosine | -0.158 | -0.001 | -0.149 | 0.004 | **0.303^*^** | **0.338^**^** | 0.236 | **0.256^*^** | 1 |
| BMI | -0.226 | **-0.4^**^** | -0.045 | **-0.312^*^** | 0.024 | 0.031 | 0.046 | 0.205 | -0.049 |
| WHR | **0.259^*^** | 0.016 | 0.218 | -0.047 | 0.094 | 0.086 | 0.116 | 0.239 | -0.091 |
| Glucose | -0.011 | -0.156 | 0.043 | -0.135 | **0.334^**^** | 0.194 | **0.398^**^** | **0.433^***^** | 0.125 |
| Insulin | 0.265 | -0.005 | 0.268 | -0.088 | 0.121 | 0.129 | 0.092 | **0.251^*^** | 0.1 |
| HOMA-IR | 0.195 | -0.016 | 0.198 | -0.073 | 0.141 | 0.182 | 0.174 | **0.307^*^** | 0.113 |
| UA | 0.117 | -0.209 | **0.258^*^** | -0.236 | 0.179 | 0.223 | **0.268^*^** | **0.358^**^** | 0.023 |
| Cholesterol | 0.032 | -0.107 | 0.116 | -0.143 | **0.351^**^** | 0.117 | 0.122 | **0.453^***^** | **0.257^*^** |
| LDL | 0.118 | **-0.326^*^** | **0.271^*^** | **-0.417^**^** | **0.296^*^** | -0.07 | -0.055 | **0.47^***^** | 0.173 |
| HDL | 0.055 | 0.126 | 0.009 | 0.053 | 0.255 | -0.101 | -0.004 | 0.072 | 0.078 |
| TG | 0.025 | -0.213 | 0.098 | -0.168 | 0.27 | **0.366^**^** | 0.009 | **0.335^**^** | 0.034 |
| WBC | -0.004 | -0.162 | 0.073 | -0.118 | 0.196 | 0.028 | -0.174 | 0.169 | -0.041 |
| CRP | 0.065 | -0.1 | 0.104 | -0.043 | 0.241 | 0.133 | -0.013 | **0.321^*^** | 0.02 |
| Fibrinogen | 0.092 | -0.016 | 0.064 | -0.062 | **0.252^*^** | 0.208 | -0.021 | **0.291^*^** | 0.034 |
| SBP | 0.212 | -0.017 | 0.212 | -0.033 | **0.366^**^** | 0.259^*^ | 0.026 | **0.359^**^** | 0.23 |
| DBP | 0.04 | -0.229 | 0.112 | -0.21 | -0.022 | 0.127 | -0.041 | 0.229 | -0.085 |
